# Hybrid Positron Emission Tomography and Magnetic Resonance Imaging Guided Microsurgical Management of Glial Tumors: Case Series and Review of the Literature

**DOI:** 10.3390/diagnostics14141551

**Published:** 2024-07-18

**Authors:** Yusuf Sukru Caglar, Murat Buyuktepe, Emre Yagiz Sayaci, Ihsan Dogan, Melih Bozkurt, Elif Peker, Cigdem Soydal, Elgin Ozkan, Nuriye Ozlem Kucuk

**Affiliations:** 1 Department of Neurosurgery, Ankara University School of Medicine, 06230 Ankara, Turkey; scaglar@ankara.edu.tr (Y.S.C.); emresa89@gmail.com (E.Y.S.); ihsandogan@ankara.edu.tr (I.D.); 2 Department of Neurosurgery, Unye State Hospital, 05230 Ordu, Turkey; 3 Department of Neurosurgery, Memorial Bahcelievler Hospital, 34180 Istanbul, Turkey; melihbozkurt@hotmail.com; 4 Department of Radiology, Ankara University School of Medicine, 06230 Ankara, Turkey; epeker@ankara.edu.tr; 5 Department of Nuclear Medicine, Ankara University School of Medicine, 06230 Ankara, Turkey; csoydal@ankara.edu.tr (C.S.); eozkan@ankara.edu.tr (E.O.); okucuk@medicine.ankara.edu.tr (N.O.K.)

**Keywords:** hybrid imaging, magnetic resonance imaging, positron emission tomography, radiation necrosis, recurrent glial tumor

## Abstract

In this case series, we aimed to report our clinical experience with hybrid positron emission tomography (PET) and magnetic resonance imaging (MRI) navigation in the management of recurrent glial brain tumors. Consecutive recurrent neuroglial brain tumor patients who underwent PET/MRI at preoperative or intraoperative periods were included, whereas patients with non-glial intracranial tumors including metastasis, lymphoma and meningioma were excluded from the study. A total of eight patients (mean age 50.1 ± 11.0 years) with suspicion of recurrent glioma tumor were evaluated. Gross total tumor resection of the PET/MRI-positive area was achieved in seven patients, whereas one patient was diagnosed with radiation necrosis, and surgery was avoided. All patients survived at 1-year follow-up. Five (71.4%) of the recurrent patients remained free of recurrence for the entire follow-up period. Two patients with glioblastoma had tumor recurrence at the postoperative sixth and eighth months. According to our results, hybrid PET/MRI provides reliable and accurate information to distinguish recurrent glial tumor from radiation necrosis. With the help of this differential diagnosis, hybrid imaging may provide the gross total resection of recurrent tumors without harming eloquent brain areas.

## 1. Introduction

Gliomas are the most common malignant tumors of the brain, accounting for approximately 80% of all cancers in the central nervous system, and the incidence has risen in recent decades with improved diagnostic imaging [1,2]. The main differential diagnoses of gliomas are lymphoma, metastasis and inflammatory/infectious diseases. The standard care of high-grade gliomas comprises early detection and maximum safe surgical resection followed by postoperative chemoradiotherapy [3]. Despite the advancements in diagnosis and treatment, the survival of those with brain tumors still remains poor [4,5]. In addition to patient characteristics including age and Karnofsky score, the histological subtype of tumor, the level of differentiation and extent of tumor removal are important prognostic factors [5,6,7,8]. Since the interaction of tumors with eloquent brain areas mainly determines the surgical approach and, thereby, extent of tumor removal, there have been increasing demands on preoperative imaging methods revealing overall extensions of tumor tissue. It is also important to evaluate the presence of residual tumor tissue intraoperatively, as well as to differentiate tumor recurrence from pseudorecurrence and radiation necrosis in the follow-up period.

Magnetic resonance imaging (MRI) and positron emission tomography (PET) are well-established imaging modalities that are widely used in neuro-oncology. However, considering brain tumors with structural, functional or metabolic information only is inadequate for pathologic estimation and treatment planning. As both techniques are complementary and essential for diagnosis and follow-up, hybrid multimodal imaging with integrated PET/MRI might have the potential to improve glioma management [9].

In this preliminary study, we aimed to report our experience with hybrid PET/MRI navigation in the management of recurrent brain tumors.

## 2. Materials and Methods

This study was approved by the IRB Committee of the Ankara University (Date: 21 May 2021, Number: I5-318-21). Written informed consent was obtained from each patient before enrollment, and this study adhered to the tenets of the Declaration of Helsinki.

In this case series study, consecutive high-grade glioma patients who had been treated with total or gross total resection surgery followed by chemoradiotherapy were evaluated. Patients with a high index of suspicion for glioma recurrence during the follow-up period who also underwent a hybrid PET/MRI scan at our hospital from October 2018 to May 2021 were included in the study. Patients with non-glial intracranial tumors including metastasis, lymphoma and meningioma were excluded from the study. Hybrid images were used for differential diagnosis, treatment planning and intraoperative guidance.

All patients underwent PET/MRI on a fully integrated General Electric system Signa PET/MRI with a head coil (General Electric Healthcare, Chicago, IL, USA). This system is equipped with a 3-T magnet and a high-resolution PET detector, integrated with Time-Of-Flight (TOF) technology, and provides the simultaneous acquisition of PET and MRI data [10]. The PET/MRI scans were performed 45 min after the intravenous administration of ^18^F-fluorodeoxyglucose (FDG), during which the patients rested in a quiet room. At least 6 h of fasting was required prior to its administration. The patients were placed in the scanner, and contiguous transaxial slices were obtained.

For PET/MRI neuronavigation, all the patients included in the study had T2-weighted propeller axial images (TR: 3570, TE:110, FOV: 240, slice thickness/gap:5/0.5), sagittal 3D FLAIR images (TR > 6000, TE:105, TI: 1500–1800, FOV: 260, slice thickness/gap:1.2/0) and pre- and post-contrast sagittal 3DT1WI BRAVO images (3D, TR:8, TE:3, FOV: 260, slice thickness/gap:1.2/0). For contrast-enhanced imaging, gadobutrol (Gadovist, Bayer Schering Pharma, Berlin, Germany) or gadoterate meglumine (dotarem; Guerbet, Aulnay-sous-Bois, France) was administered at a single dose of 0.1 mmol/kg by intravenous bolus injection at a rate of 2 mL/s.

Data were described as the mean ± standard deviation for numerical and frequency (percentage) for categorical variables. Statistical analyses were performed with the Statistical Package for Social Sciences (SPSS Version 24.0, Chicago, IL, USA).

## 3. Results

Eight patients (five males), with a mean age of 50.1 ± 11.0 years (range, 28–62 years), were included in the study. Table 1 shows the clinical and demographic data of patients.

All patients underwent uneventful neurosurgical procedures and did not encounter any neurological deficit thereafter. Hybrid PET/MRI images were used for surgical planning and intraoperative guidance in tumor resection. Gross total tumor resection of the PET/MRI-positive area was achieved in all patients, except case 1, whose PET/MRI was reported as radiation necrosis. The extent of resection was confirmed with postoperative CT or MRI in all seven patients.

A postoperative histopathological investigation of the tumors revealed recurrent glioma (Table 1). All patients survived at 1-year follow-up. In case 1, the diameter of the radiation necrosis area was stable, and no new neurological symptoms were encountered at 1-year follow-up. Five (71.4%) patients remained free of recurrence for the entire follow-up period. Two patients with glioblastoma had tumor recurrence at the postoperative sixth and eighth months.

### 3.1. Illustrative Cases: Case 3

**Figure 1:**
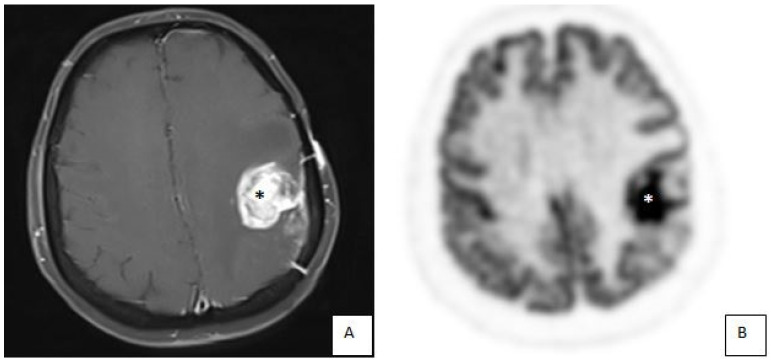
Case 3. Magnetic resonance imaging (MRI) (**A**) revealed an enhancing mass lesion (black asterisk) in the left frontoparietal lobe. A hybrid PET/MRI image (**B**) showed a hot spot (white asterisk), suggesting recurrent glioma.

A 58-year-old female patient applied to our hospital with a complaint of dysarthria for 1 week. She had been operated on once for intracranial tumor previously, and the postoperative histologic diagnosis was high-grade glioma, followed by radiochemotherapy and adjuvant chemotherapy with Temozolamide. Cranial MRI revealed an enhancing mass lesion in the left frontoparietal lobe, whereas MR spectroscopy (MRS) suggested radiation necrosis. PET/MRI showed an F-^18^FDG hot spot suggesting recurrent glioma (Figure 1). The patient underwent gross total resection using hybrid PET/MRI neuronavigation. Hybrid images provided data to delineate tumor margins and resection borders with high accuracy. Histopathological evaluation confirmed the diagnosis of recurrent glioma.

### 3.2. Illustrative Cases: Case 5

**Figure 2:**
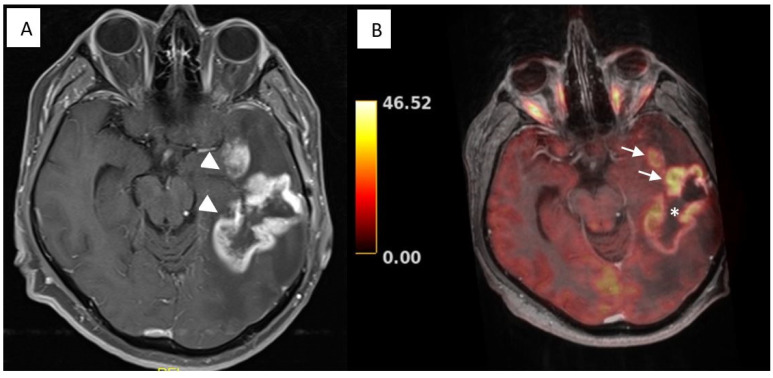
Case 5. T1-weighted magnetic resonance imaging (MRI) (**A**) revealed heterogenous contrasting lesions (arrowheads). Positron emission tomography/MRI images (**B**) showed focal hot spots (arrows) within the previously operated area (asterisk) as well as in a different area.

In a 53-year-old male patient with a complaint of aphasia and orobuccal seizure, a recurrent suspicious contrast-enhancing lesion within his temporal lobe occurred 8 months after GBM resection. T2W MRI showed a hyperintense mass lesion containing cystic and necrotic areas. After contrast administration, the lesion enhanced heterogeneously. PET/MRI images showed focal hot spots within the previously operated area as well as in a different area (Figure 2). Hybrid images helped to distinguish between recurrence and postradiation effects and optimized re-resection. Gross total tumor removal was achieved with hybrid neuronavigation, and histologic examination revealed glioblastoma, which was suggested as postradiation necrosis on MRI images. At postoperative month 6, MRI images revealed a hyperintense mass lesion, suggesting tumor recurrence, but the patient refused re-treatment.

## 4. Discussion

According to our results, imaging is very helpful for the differential diagnosis of tumor recurrences from treatment-related changes, as well as the surgical planning of neuroglial brain tumors.

Neurosurgical imaging systems are important in neuro-oncology, and they are continuously improving in diagnostic performance and patient comfort. While MRI is the most widely used tool for the diagnosis and follow-up of high-grade glioma, the reliability of the conventional MRI series is limited in the determination of treatment response or tumor progression due to potential treatment-induced signal changes [11,12,13]. One of the diagnostic dilemmas in the follow-up period of high-grade glioma cases is pseudoprogression, which is defined as an enlarging or new lesion appearing on MRI after the concurrent administration of radiotherapy and chemotherapy (i.e., temozolamide) but without any true progression. It is attributed to radiation necrosis and inflammatory changes or treatment-related alterations in the blood–brain barrier, leading to increased vascular permeability [14]. It is seen in up to 36% of high-grade glioma [15,16]. There is an increasing demand for definitive radiological criteria for a differential diagnosis of pseudoprogression and true progression to prevent any invasive biopsy or premature cessation of efficacious therapeutic agents. Pseudoresponse, on the other hand, is a rapid resolution of focal enhancement on MRI without a true remission of the tumor and is mostly caused by the anti-angiogenic effect of Bevacizumab [17,18].

In post-treatment high-grade glioma cases, the determination of metabolic activity in enhanced areas on MRI is crucial for the differential diagnosis of true progression or true response. Advanced MRI sequences, including perfusion MRI and MRS, provide more metabolic information and thus better diagnostic accuracy in recurrent glioma cases compared to conventional MRI [18]. Restricted diffusion and an elevated relative cerebral blood volume are indicative for true progression, rather than pseudoprogression [19]. But the cut-off values of PWI are variable in the reported studies, and clinical experience is limited with MRS [18]. As yet, no single technique can be regarded as a gold standard. Nevertheless, more precise information about the metabolic activity of tumor tissue can be obtained by PET imaging. It allows for the combination of multiple diagnostic data, such as tumor blood volume and glucose uptake with the help of radioactive substances. However, it is still limited by low spatial and temporal resolution [20]. There is a need to develop new modalities integrating anatomical, functional and biological information, leading to extremely accurate diagnostic examination and surgical planning. The hybrid PET/MRI scanner is the first implementation of two modalities, in which PET photons and MR signals are co-registered in multimodal images. It allows for the combination of the high contrast and morphological resolution of MRI with the metabolic and physiological information from the integrated PET scan in a single session [21].

There are three different options for combining PET and MRI data: (i) the retrospective fusion of separate PET and MRI images; (ii) sequential/co-planar PET/MRI, during which the patient remains positioned on the same table and travels from PET to MRI; and (iii) integrated hybrid PET/MRI, which provides the simultaneous acquisition of data [22]. There are a number of technical factors limiting the accuracy of images in all combined PET/MRI protocols, but simultaneous PET/MRI provides the best image fusion with a better spatial and temporal resolution. Besides its technical and clinical advantages, obtaining PET and MRI images simultaneously provides decreased examination time and increased comfort for often severely ill patients [23]. Both retrospective fusion and sequential PET/MRI protocols have a longer scanning time compared to hybrid PET/MRI, which may also lead to serious artifacts from patients’ voluntary movements due to long-lasting imaging [21].

In the previous literature, Jena et al. prospectively evaluated 26 malignant glioma cases with hybrid PET/MRI to assess glioma recurrence versus radiation necrosis and reported that the highest diagnostic accuracy (96.9%) was achieved by a combined analysis of PET and MRI parameters such as the mean target-to-background ratio and choline-to-creatinine value [24]. Similarly, Sogani et al. conducted a prospective study on 32 consecutive glioma patients with suspicions of recurrence using integrated PET/MRI and again demonstrated that the combination of PET and MRI parameters improved the predictive value for the differentiation of true progression from treatment-related changes, compared to any single parameter. The reported diagnostic accuracy, sensitivity and specificity of integrated analysis were as high as 96.87%, 100% and 85.7%, respectively [25]. Furthermore, Pyka et al. evaluated 63 lesions suggestive of glioma recurrence and performed a dynamic PET scan, as well as morphologic MRI, perfusion MRI and diffusion MRI on the hybrid PET/MRI scanner [26]. They reported that a multiparametric analysis of PET and MRI metrics provided synergistic value for the differential diagnosis of glioma progression, with 76% sensitivity and 100% specificity [26]. In our series, hybrid PET/MRI, morphological MRI, MRS or perfusion MRI images were compared in high-grade glioma patients. While all modalities were to some extent able to discriminate between progression and pseudoprogression, hybrid PET/MRI outcomes were highly correlated with a histopathological diagnosis of glioma recurrences. Indeed, these were more reliable than MRS outcomes. Hybrid PET/MRI and MRI techniques were congruent in terms of tumor size, since both techniques demonstrated an MRI-based structure.

To date, different radiopharmaceuticals for PET scans have been used in neuro-oncology [27]. FDG is well known and the most widely available PET tracer. It indicates glucose uptake and metabolism and thus differentiates low- from high-grade gliomas. Its uptake is increased in high-grade gliomas, whereas well-differentiated neuroepithelial tumors exhibit a low level of accumulation due to a low level of glycolysis [28]. FDG uptake was also evaluated for the assessment of the isocitrate dehydrogenase (IDH) genotype and thereby for the prediction of prognosis in glioma patients [29]. Although it is widely used for tumor grading and biopsy planning, non-specific FDG uptake by normal brain tissue or during inflammation is the main disadvantage. On the other hand, radiolabeled amino acids (i.e., ^18^F-deoxyphenylalanine; ^18^F-fluoroethlythyrosine, F-FET; ^11^C-methionine, C-MET; ^18^F-fluoro-L3,4-dehydroxyphenylalanine, F-FDOPA) indicate amino acid uptake and protein synthesis. These tracers are recommended by international guidelines to complement MRI in the clinical management of patients with gliomas [30,31]. Several studies investigated the potential of amino acid PET tracers for the diagnosis of molecular markers of gliomas, including the IDH genotype, 1p/19q codeletion and O-methyl guanine methyltransferase (MGMT) promoter methylation status [32,33,34,35]. In addition, these tracers are more reliable than FDG in a postradiation assessment of recurrences, due to relatively less uptake by inflammation. F-FET has been shown to differentiate the recurrence of brain metastases from treatment-related changes with high accuracy [36,37], and C-MET PET and structural MRI images were used to develop a reliable model for distinguishing recurrent brain tumor from radiation necrosis [38,39]. Recently, an artificial amino acid tracer, anti-1-amino-3-^18^F-fluorocyclobutane-1-carboxylic acid (^18^F-FACBC), was defined in brain tumors; increased FACBC uptake was demonstrated in high-grade gliomas, compared to normal brain tissue [40,41]. In a study conducted on recurrent glioma cases, the tumor uptake of ^18^F-FACBC was reported to be correlated with C-MET but provide significantly higher image contrast [42]. Another group of tracers are choline-labeled PET tracers (^11^C-choline, ^18^F-fluorocholine), which are markers for lipid metabolism and plasma membrane turnover. They have the advantages of better tumor delineation than other tracers due to the very low uptake by normal brain tissue [27,43]. Lastly, prostate-specific membrane antigen (PSMA), which is a transmembrane glycoprotein and highly expressed in prostate cancer, was found to be expressed in high-grade gliomas due to tumor neovascularization [44,45]. In the setting of the suspicious recurrence of high-grade gliomas, PSMA uptake was demonstrated to be significantly higher among tumor recurrences, compared to radiation necrosis [46]. Although there is a strong concordance between FDG and PSMA uptake in the initial diagnosis of high-grade gliomas and evaluation of tumor recurrences, PSMA-targeting radiopharmaceuticals were found to be more accurate than FDG, due to the absence of physiological radiopharmaceutical uptake in normal brain parenchyma [47,48]. Overall, in recent studies using PET/MRI for the assessment of glioma recurrence, the F-FET tracer has been utilized most [49]. However, in our study, we were only able to use the FDG tracer during PET scans due to local availability. Even so, FDG-PET/MRI was found to be effective in terms of the differentiation of radiation necrosis from progression.

In addition to recent advancements in neuroimaging, the use of fluorescence agents such as 5-aminolevulinic acid (5-ALA), indocyanine green (ICG), or sodium fluoresceine allows for a further visualization of tumoral tissue and has been demonstrated to maximize the extent of resection intraoperatively [50]. Furthermore, Barbagallo et al. evaluated the extent of resection in recurrent gliomas using multimodal imaging with intraoperative CT, MRI, PET, ultrasonography and fluoroscopy [51]. They reported increased safety and efficacy with recurrent high-grade glioma and brain alterations secondary to radiochemotherapy [51]. Fluorescence guidance helped to discriminate tumoral and non-tumoral changes during surgery, whereas PET/MRI was used to differentiate recurrences during preoperative surgical planning.

There are several limitations in our study, including a limited number of patients. This limits the generalizability of our findings. Still, a histopathological evaluation of PET/MRI-positive areas was performed in all patients and thereby enhanced the quality of interpretation of hybrid PET/MRI outcomes. We believe the results of our cases may be important to understanding the clinical role of PET/MRI in differential diagnosis as well as surgical planning.

In conclusion, the hybrid PET/MRI of recurrent glial tumors could increase diagnostic accuracy in the prediction of disease progression and play a game-changing role in the management of high-grade glioma patients. Further prospective studies with a larger number of patients may help to establish the diagnostic value and clinical implementation of new hybrid imaging techniques.

## Figures and Tables

**Table 1:** Demographical and clinical features of patients.

Case	Age (Years)	Sex	Histological Diagnosis of Primary Tumor	Localization	Size (cm)	Tracer	Surgery
1	59	M	Glioblastoma	L Frontal	2.3 × 3.5 × 2.8	FDG	None
2	48	M	Diffuse Astrocytoma	L Frontal	6.9 × 4.8 × 4.6	FDG	Gross total resection
3	58	F	Glioblastoma	L Frontoparietal	3.0 × 3.2 × 3.4	FDG	Gross total resection
4	45	M	Gemistocytic Astrocytoma	L Frontoparietal	2.5 × 2.0 × 1.9	FDG	Gross total resection
5	53	M	Glioblastoma	L Temporal	2.0 × 2.0 × 2.3	FDG	Gross total resection
6	47	F	Oligodendroglioma	R Temporal	3.3 × 3.4 × 2.6	FDG	Gross total resection
7	28	M	Glioblastoma	R Hippocampus	6.0 × 4.5 × 4.5	FDG	Gross total resection
8	63	F	Glioblastoma	L Frontoparietal	5.4 × 4.7 × 3.7	FDG	Gross total resection

Abbreviations: F: female, FDG: fluorodeoxyglucose, L: left, M: male, R: right.

## Data Availability

The data presented in this study are available on request from the corresponding author. The data are not publicly available due to the ethical reasons.
